# Adaptive differentiable grids for cryo-electron tomography reconstruction and denoising

**DOI:** 10.1093/bioadv/vbad131

**Published:** 2023-09-22

**Authors:** Yuanhao Wang, Ramzi Idoughi, Darius Rückert, Rui Li, Wolfgang Heidrich

**Affiliations:** Visual Computing Center (VCC), King Abdullah University of Science and Technology (KAUST), Thuwal 23955-6900, Saudi Arabia; Visual Computing Center (VCC), King Abdullah University of Science and Technology (KAUST), Thuwal 23955-6900, Saudi Arabia; Department of Computer Science, Friedrich-Alexander-Universität Erlangen-Nürnberg (FAU), 91054 Erlangen, Germany; Visual Computing Center (VCC), King Abdullah University of Science and Technology (KAUST), Thuwal 23955-6900, Saudi Arabia; Visual Computing Center (VCC), King Abdullah University of Science and Technology (KAUST), Thuwal 23955-6900, Saudi Arabia

## Abstract

**Motivation:**

Tilt-series cryo-electron tomography is a powerful tool widely used in structural biology to study 3D structures of micro-organisms, macromolecular complexes, etc. Still, the reconstruction process remains an arduous task due to several challenges: The missing-wedge acquisition, sample misalignment and motion, the need to process large data, and, especially, a low signal-to-noise ratio.

**Results:**

Inspired by the recently introduced neural representations, we propose an adaptive learning-based representation of the density field of the captured sample. This representation consists of an octree structure, where each node represents a 3D density grid optimized from the captured projections during the training process. This optimization is performed using a loss that combines a differentiable image formation model with different regularization terms: total variation, boundary consistency, and a cross-nodes non-local constraint. The final reconstruction is obtained by interpolating the learned density grid at the desired voxel positions. The evaluation of our approach using captured data of viruses and cells shows that our proposed representation is well adapted to handle missing wedges, and improves the signal-to-noise ratio of the reconstructed tomogram. The reconstruction quality is highly improved in comparison to the state-of-the-art methods, while using the lowest computing time footprint.

**Availability and implementation:**

The code is available on Github at https://github.com/yuanhaowang1213/adaptivediffgrid_ex.

## 1 Introduction

Tilt-series cryo-electron tomography (cryo-ET) has become an increasingly popular technique in structural biology, used to reconstruct the 3D structures of micro-organisms and macromolecular complexes with a high resolution. This technique involves collecting a series of projections (images) of the sample from different angles. Then, tomographic reconstruction tools are applied to generate 3D tomogram of the scanned sample. Despite the advances in technologies and software (Bai *et al.* 2015), the tomographic reconstruction of tilt-series remains a challenging task for several reasons, such as: missing-wedge acquisition, extensive data size, high noise levels, misalignment of projections, and sample motion during the acquisition process. In this work, we propose an approach that handles the first three problems, while projections alignment and correction are performed as a preprocessing step.

The missing-wedge acquisition induces a lack of angular information, which results in artifacts and limited axial resolution in the final reconstruction. In addition, cryo-ET reconstruction requires the processing of a large amount of data, which can be time consuming and computationally intensive. Moreover, cryo-ET acquisition involves only limited-dose electron beams to avoid potential damage to the samples by electrons. This causes severe noise in the captured projections (Radermacher 1992). Denoising becomes an essential task in cryo-ET processing. Hence, several models have been explored in the literature for the cryo-ET noise, such as the additive white Gaussian noise (Bepler *et al.* 2020), or a Poisson–Gaussian noise (Zhang *et al.* 2019b). However, when electrons hit the sensors, they may be detected by several pixels at the same time. This electron spread makes the actual noise more complex to model.

In most existing approaches, denoising is applied either before or after the reconstruction step (Frangakis 2021), which is performed using classical algorithms like Weighted Filtered Back-Projection (WFBP) (Radermacher 2007). Several denoising algorithms from the computer vision field have been applied to cryo-ET reconstruction, such as the bilateral filter (Jiang *et al.* 2003), the non-local means filtering (Wei and Yin 2010), the wavelet shrinkage filter (Huang *et al.* 2018), and deep learning-based techniques (Buchholz *et al.* 2019). Total variation (TV) (Zhang and Bajaj 2010) and volume-based non-local transform-domain filter methods like BM4D (Maggioni *et al.* 2013) were the state of the art in tomograms denoising, for a decade. However, these approaches require considerable computational resources.

Learning-based methods gained great success in denoising tasks. Specifically, unsupervised methods, such as Noise2Noise (Lehtinen *et al.* 2018) and Noise2Void (Krull *et al.* 2019), present an excellent potential for tomograms denoising, since there are no ground truth data in the cryo-ET reconstruction field to be used for the learning step. Bepler *et al.* (2020) proposed the Topaz algorithm that leverages the Noise2Noise concept to produce clean tomograms. In their approach, two tomograms are reconstructed from odd/even projections and used for the learning step as a pair of noisy data instead of paired noisy and ground truth data. This approach assumes an independent and homogeneous noise with a zero mean. Li *et al.* (2022) proposed to learn the noise distribution directly from the pure noise patches in the projections, in order to restore the signal and enhance the image contrast.

Drawing on the impressive and rapid advancements in neural representations, also known as coordinate-based neural networks, in recent years (Tewari *et al.* 2022, Xie *et al.* 2022), we propose a new learning-based representation suitable for the large and noisy data in cryo-ET, allowing a joint reconstruction and denoising of the scanned samples. Neural representation approach was first introduced by Mildenhall *et al.* (2020) with their so-called Neural Radiance Field (NeRF). They proposed to train multi-layer perceptron (MLP) networks to represent 3D scenes’ physical properties (e.g. density field and color) as continuous volumetric functions. These networks get the spatial coordinates and, eventually, the viewing directions as inputs. Then, they are trained using a sparse set of captured data. Specifically, a set of 3D points is sampled along a given ray. Then, the coordinate-based networks are evaluated at these positions. Finally, the integration of all these contributions is compared to captured data in the loss function, in order to update the coordinate-based network.

These neural fields have been extensively applied to handle different tasks in the computer vision and graphics fields (Tewari *et al.* 2022, Xie *et al.* 2022). Neural fields have also been applied for Computed Tomography reconstruction problem (Sun *et al.* 2021, Zang *et al.* 2021, Rückert *et al.* 2022). They yielded impressive results even in the challenging missing-wedge configuration that is encountered in tilt-series tomography. The main advantage of neural fields approaches is their unsupervised learning strategy, where only the captured projections are needed. Moreover, the neural representation plays the role of an additional regularizer to the tomography problem, which is able to predict missing angular information. In addition, neural fields have a much lower memory requirement than classical tomographic reconstruction techniques, as they do not require the storage of the entire 3D volume during the optimization process. Nevertheless, coordinates-based networks suffer from very long training times and a slow rendering since the network must be evaluated at each sample along each ray (see Fig. 2a). Different techniques have been applied to handle this issue, including the use of octree structures (Liu *et al.* 2020, Fridovich-Keil *et al.* 2022, Rückert *et al.* 2022) and multi-scale network architecture (Martel *et al.* 2021), network factorization (Reiser *et al.* 2021), caching (Garbin *et al.* 2021), and multi-resolution hash encoding (Müller *et al.* 2022). These techniques allow for faster training and larger reconstructed data than basic MLP-based neural fields. Still, they remain inadequate to reconstruct cryo-ET data, because of the high level of noise. These networks are not designed to separate the noise from the data signal. They either learn the noise as part of the signal or oversmooth the signal in such a way to lose the main features of the data. We will illustrate this aspect in Section 3 through a comparison against the approach **NeAT** (Rückert *et al.* 2022). Kniesel *et al.* (2022) proposed to jointly learn a model for 2D sensor noise and a 3D implicit neural representation of the scanned sample. However, this approach depends on the noise level, as we will show later in the comparison.

In this work, we propose a new framework for cryo-ET reconstruction, where we jointly reconstruct and denoise the tomograms using adaptive density grids based on an octree structure. During the optimization of our proposed neural representation, nodes that compose the octree structure will be divided into new nodes, merged, or disabled if empty. This octree update is aiming to accelerate the computations by giving more importance to regions with more features. Each octree node is featured with a differentiable density grid, that we optimize from the captured noisy projections, as illustrated in Fig. 2. Our work borrows from recent advances in machine learning by optimizing the adaptive representation using a differentiable image formation model in combination with backpropagation. We show experimentally that this combination of a classical hierarchical data structure with differentiable image formation and error backpropagation outperforms recent neural representations on cryo-ET reconstruction.

To tackle the heavy noise present in the tomograms obtained in cryo-ET, we combine a multi-scale octree-update strategy with TV prior and a cross-nodes non-local constraint (CNLC) during the density update. We also use a boundary consistency prior to ensure continuity between adjacent octree nodes. The evaluation of our approach on two real datasets reveals considerable improvements over state-of-the-art reconstruction methods.

## 2 Methods

### 2.1 General overview

**Figure 1.**
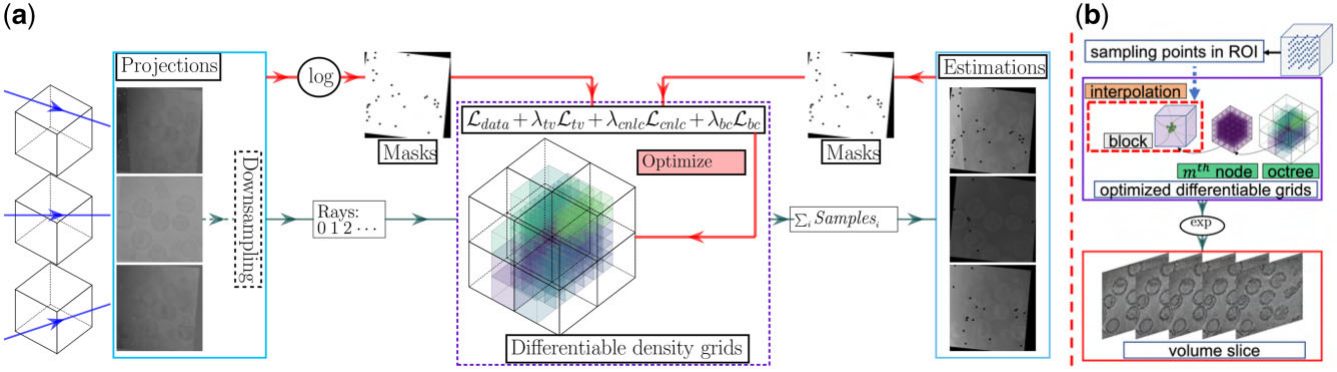
Our workflow contained two steps, the first is the training part to generate continuous differentiable density grids, and the second part is to query the densities in the ROI. (a) Adaptive density grid optimization. We use a coarse-to-fine strategy to update the density grid, which reduces the impact of noise. First, we use the down-sampled projections to update the octree structure. Then, we freeze the octree structure and update the density grid in each octree node using the original noisy projections. During this second step, we introduce masks to eliminate gray regions in the borders [padded by IMOD (Mastronarde and Held 2017) after the alignment step], as well as the markers. (b) Volume querying. After a uniform 3D sampling inside the ROI, the density is estimated at the selected positions.

Our proposed framework, illustrated in Fig. 1, is mainly based on adaptive grids representation of the scanned object that we implemented in a multi-scale hierarchical fashion. This structure is updated and optimized to represent a continuous 3D density field. The reconstruction is then obtained by uniformly sampling the volume in the region of interest (ROI) and querying the density at those samples’ positions. The loss function used in the training step is composed of a data-fidelity term derived from the tomography formation model, and three different regularization terms to improve the reconstruction quality.

In the following, we first introduce the image formation model, and the different constraints incorporated into our loss function: the TV, the non-local constraint, and the boundary consistency. Then, we present the adaptative differentiable grids representation of the volume, the sampling strategy, and the model optimization.

### 2.2 Image formation model

For a tomographic reconstruction, the projection image captured by the sensor corresponds to the integration in the log space of density along the rays between the source and the different detectors. For a given ray, the formation model can be written after discretization using the following equation:


(1) $$b_{i}=A_{i}x_{i}+n_{i},$$

where $b_{i}$ and $n_{i}$ correspond, respectively, to the intensity measured by the detector *i* and its associated noise. $A_{i}$ stands for the Radon transform operation along the ray *i*. $x_{i}$ is a vector of 3D density values sampled along the ray. In cryo-ET tilt-series, the noise is frequently assumed to follow a Poisson noise model in the raw captured data (Anoshina *et al.* 2018). However, after several preprocessing operations, such as the motion correction and the intensity correction, the noise in the projections becomes more intricate and can be considered as Gaussian noise for the sake of simplicity. By regrouping all rays together and applying a mask to disable the rays that intersect with a fiducial marker, we define the following data-fidelity loss:


(2) $$L_{\mathrm{data}}(x)=\frac{1}{2}||M\left(Ax-b\right)|{|}_{2}^{2},$$

where M is a binary mask used to limit the optimization process to rays that do not intersect fiducial markers. Details on the generation of M are provided in the Supplementary Material.

### 2.3 Coordinate-based representation

**Figure 2.**
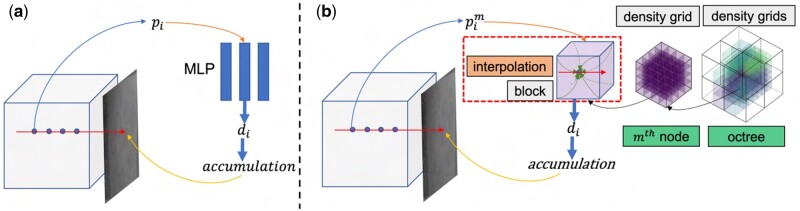
We represent in (a) the application of NeRF to the tomography reconstruction, while in (b), we depict the general idea of our approach. We utilize an adaptive differentiable density grids for replacing the MLP for a faster optimization and a better feature recovering.

The application of coordinate-based networks to tomography problems consists of mapping the 3D spatial coordinates inside the ROI to the density field. This mapping can be expressed as:


(3) $$f_{\phi }:p_{i}\to x_{i}\;\text{with}\;p_{i}\in R^{3},x_{i}\in R,$$

where $p_{i}$ represents the 3D coordinate in the volume, and $x_{i}$ is the corresponding density. $f_{\phi }$ stands for the representing function to be optimized. In traditional neural fields, $f_{\phi }$ is a fully connected MLP as shown in Fig. 2a. This representation quickly comes up against a limitation due to the size of the reconstructed scene. That is why several follow-up works, such as ACORN (Martel *et al.* 2021) and NeAT (Rückert *et al.* 2022) proposed using a multi-scale structure based on octrees. In these approaches, each octree node has a smaller MLP or a decoder network to represent locally the densities. Our framework, illustrated in Fig. 2b, uses a similar representation based on adaptive octree structure. However, in our representation, each octree node stores an optimizable 3D density grid representing locally and in a discretized manner the mapping function $f_{\phi }$. For a given 3D point p, its density is obtained by a trilinear interpolation from the stored densities of the eight vertexes of the block of the density grid that contains p, as shown in the red dashed rectangle in Fig. 2b. Since the interpolation operation is differentiable, it is possible to optimize the proposed 3D density representation. Thus, the data-fidelity loss function could be rewritten as:


(4) $$L_{\mathrm{data}}(\phi )=\frac{1}{N}\sum \left|\right|M\left(Af_{\phi }(p)-b\right)|{|}_{2}^{2},$$

where *N* is the number of sampling points (batch size) used at each iteration of the training step. For the sake of simplification, we define the same size for all density grids $N_{x}\times N_{y}\times N_{z}$ (in this article, $N_{x}=N_{y}=N_{z}$). $N_{x}$, $N_{y}$, and $N_{z}$ represent the grid size in *x*, *y*, and *z* dimension, respectively.Our representation has several advantages over the existing representations. First, in most cryo-ET datasets, some regions of the scanned sample have detailed features, while others are uniform. By using multi-scale octree structure to represent these different regions, our reconstruction will have a better saving of the details in the sample while denoising the uniform regions. Moreover, the use of interpolation to query densities is much faster than using an MLP or a decoder network. Furthermore, the density grid representation allows more flexibility in adding regularizations to the loss function, in order to deal with the high level of noise in cryo-ET data.

### 2.4 Regularizations

To reduce the noise level in our reconstruction and to ensure that the neural field is continuous at the grid edges, we introduce three different priors in our loss function.

#### 2.4.1 Total variation

The first prior term in our framework is the TV loss, commonly used in tomographic reconstruction as a spatial regularizer to smoothen the reconstruction. The use of this prior in neural fields is not straightforward, because it would require querying the complete volume at each iteration to evaluate the TV loss. Zang *et al.* (2021) utilized neural fields only for estimating the missing-wedges projections. Then, they introduced a TV loss in a classical reconstruction with a completed sinogram. Rückert *et al.* (2022) proposed computing this loss in the feature space, before applying a learned decoder to get the densities. In our implementation, we apply the TV prior on the density grid of each enabled octree node and then we average over all nodes, which can be expressed as follows:


(5) $$L_{tv}=\frac{1}{N_{tv}}\sum \text{mean}\left(\left|\nabla f_{\phi }(p)\right|\right),$$

where $\nabla f_{\phi }(p)$ and $N_{tv}$ correspond, respectively, to the density gradient in each grid of the octree structure, and the number of enabled nodes.

#### 2.4.2 Cross-nodes non-local constraint

Non-local means (Buades *et al.* 2005) is a classical filtering method that averages all pixels in an image, weighted by their similarity to the target pixel. It results in better denoising than local filtering approaches. It paved the way for several other algorithms, such as BM3D (Block-Matching 3D) (Dabov *et al.* 2007), which yields solid denoising performance, and stays competitive even when compared to deep learning approaches.

Recently, Wang *et al.* (2018) introduced a non-local neural network, to improve the feature representations in 3D video classification task. Zhang *et al.* (2019a) adopted the residual non-local attention networks for several image restoration tasks, including image denoising. The proposed non-local operations aim to explore self-similarity inside the image by summarizing related sampled features from a feature grid using the concept of self-attention. The non-local operation is defined in the following equation:


(6) $$y_{i}=\mathrm{softmax}(f_{\phi }{\left(p_{i}\right)}^{T}f_{\phi }(p_{j}))f_{\phi }{\left(p_{j}\right)}^{T},$$

where $f_{\phi }(p_{i})$ and $f_{\phi }(p_{j})$ are the features sampled in the feature grid at location $p_{i}$ and $p_{j}$, and $y_{i}$ is the resulted feature after non-local operation in the location $p_{i}$.

In our implementation, it is implausible to apply directly the non-local operations on the large 3D tomograms we are reconstructing. Therefore, we propose randomly selecting two enabled nodes inside the ROI at each iteration. Then, we sample the density inside their density grids to compute the non-local operation. The non-local loss is then defined as follows:


(7) $$L_{\mathrm{cnlc}}=\sum \left|\right|s\mathrm{oftmax}(f_{\phi }{\left(p_{i}^{m}\right)}^{T}f_{\phi }(p_{j}^{n}))f_{\phi }{\left(p_{j}^{n}\right)}^{T}-f_{\phi }{\left(p_{i}^{m}\right)}^{T}||,$$

where $f_{\phi }(p_{i}^{m})$ and $f_{\phi }(p_{j}^{n})$ are the densities sampled at position $p_{i}$, and $p_{j}$ inside the density grids of nodes *m* and *n*, respectively. By taking the “softmax” operation and multiplying it with the adjoint of the density vector in density grid *n*, we estimate the similarities that may exist with the density vector in density grid *m*. The validation of this loss term could be found in the ablation study in the Supplementary Material.

#### 2.4.3 Boundary consistency

The octree structure used in our framework will inevitably introduce discontinuity artifacts in the reconstructed density field, because each node has its own density grid and the optimization is not performed consistently between the different octants. To solve this issue, we introduce a boundary consistency loss, similar to the one proposed by Rückert *et al.* (2022). For all the boundaries between different grids, we minimize the difference between the densities computed using each of the concerned density grids. The boundary consistency loss can be expressed as follows:


(8) $$L_{bc}=\sum_{(n,m)\in O_{b}}\text{mean}\left(\sum_{s\in \cap_{n,m}}|f_{\phi }{\left(p\right)}_{m}-f_{\phi }{\left(p\right)}_{n}|\right).$$

Here, $O_{b}$ refers to all pairs of neighboring octree nodes, $\cap_{n,m}$ is the set of sampling points on the boundary surface between nodes *n* and *m*, $f_{\phi }{\left(p\right)}_{m}$ and $f_{\phi }{\left(p\right)}_{n}$ correspond to the densities along the boundary, evaluated using the grids *m* and *n*, respectively.

### 2.5 General loss

By combining the data-fidelity term with the three regularizer terms discussed previously, the loss function used in our framework can be expressed as:


(9) $$L_{\mathrm{total}}=L_{\mathrm{data}}+\lambda_{tv}L_{tv}+\lambda_{\mathrm{cnlc}}L_{\mathrm{cnlc}}+\lambda_{bc}L_{bc},$$

where $\lambda_{tv}$, $\lambda_{\mathrm{cnlc}}$ and $\lambda_{bc}$ are the weighting parameters for the TV term, the CNLC, and the boundary consistency prior, respectively.

### 2.6 Adaptive density grid optimization

After defining our neural representation and the loss function used for our optimization, we present in the following the main components of the training step of our framework: the octree-update and the ray sampling.

#### 2.6.1 Octree update

**Figure 3.**
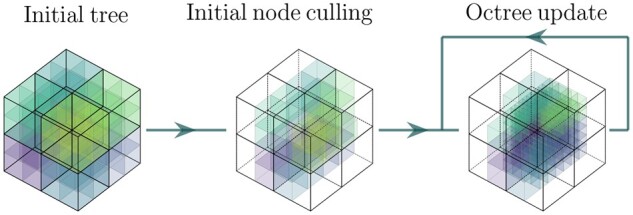
Octree update. We initialized the octree by disabling the nodes out of the ROI, and updated the tree only inside the ROI.

The first step consists of defining the octree used for our representation. First of all, the octree is initialized from the ROI, where the outsider nodes are disabled, as shown in Fig. 3. Then, we uniformly sample each octant and compute the SD of the densities inside each node (density grid), to define the update loss. When the SD in a given node is significant, it means the node is likely to contain detailed features. Thus, this node will have a higher chance of being divided into eight child octants. We follow the same octree-update constraint as ACORN (Martel *et al.* 2021) and NeAT (Rückert *et al.* 2022). That consists of solving a mixed-integer program, in which the octants are either divided, merged, or kept the same at each iteration according to the update loss, to keep the total number of nodes lower than a fixed limit. In our case, the update loss is different from those selected by ACORN and NeAT. In the first approach, the ground truth density is available in each position of the image/volume. However, in tomographic reconstructions, this is not the case.

Therefore, NeAT proposed to use reprojection errors to define the octree-update loss. Nevertheless, this turns out not to be a good option for cryo-ET datasets because of the high noise in the correspondent projections. Instead, we find that taking the SD of each node as the octree-update loss is a better option for cryo-ET datasets.

To address the high level of noise issue, we further apply a coarse-to-fine strategy to accelerate the updating of the octree. The octree is initially updated using down-sampled projections. During this step, we also optimize the densities of the density grid inside each node. The low-resolution projections allow the reduction of the impact of the high noise during the updating of the octree. After several epochs, we fix the structure of the octree, and use the original projections to optimize only the density grid of each node. Note that at this step, the grids are initialized from their down-sampled version.

#### 2.6.2 Ray sampling

**Figure 4.**
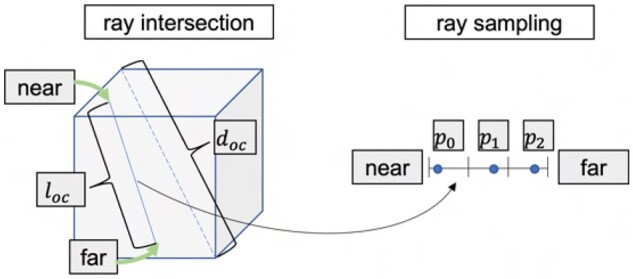
We estimate the number of the samples in the ray intersection by using Equation (10), and use a stratified sampling strategy to generate the sampling points.

During the optimizing step, each ray is sampled to define a list of 3D positions to be used in the intensity integration and loss evaluation. This sampling is not uniform, but takes into account the current octree structure. For each octree node crossed by the ray, we select $N_{oc}$ 3D positions along the ray, stratified randomly sampled (see in Fig. 4). This number $N_{oc}$ is defined as follows:


(10) $$N_{oc}=\left\lceil N_{\text{max}}\frac{l_{oc}}{d_{oc}}\right\rceil ,$$

where $N_{\text{max}}$ is a hyperparameter corresponding to the maximum number of samples per node, $l_{oc}$ is the length of the ray inside the octree node *oc*, and $d_{oc}$ is the length of the diagonal of *oc*.

## 3 Experiments and results

We designed several experiments to demonstrate the effectiveness of our framework on both simulated datasets (see Supplementary Material), and real captured datasets in the current section. We compared our approach to three main state-of-the-art tomographic reconstruction techniques: (i) **SART+TV** (Simultaneous Algebraic Reconstruction Technique + Total Variation prior)—this iterative optimization-based approach is known to provide better results than WFBP in missing-wedge acquisition (Wang *et al.* 2022), (ii) **Kniesel *et al.***method proposed by Kniesel *et al.* (2022), and (iii) **NeAT** proposed by Rückert *et al.* (2022). We also compare our approach without the cross-nodes non-local constraint (**Ours W/O CNLC**). To make a fair comparison, all the reconstructed densities are normalized into [0,1] range. We further evaluate the effectiveness of the denoising both qualitatively and quantitatively (see Section 3.3). Moreover, we propose an experiment to evaluate the performance of our approach in preserving detailed features of the scanned sample (see Section 3.4). We also provide an estimation of the reconstruction resolution using the Fourier Shell Correlation (FSC) metric (see Section 3.5). Finally, we evaluate the robustness to noise of our approach, and provide an ablation study to illustrate the importance of our framework’s components (see Supplementary Material).

### 3.1 Datasets

The datasets used in our evaluation are from the public database EMPIAR (Electron Microscopy Pilot Image Archive). Specifically, we used the tilt-series datasets indexed as: EMPIAR 10643 (Ni *et al.* 2022), EMPIAR 10453 (Turoňová *et al.* 2020), and EMPIAR 11462 (Wozny *et al.* 2023). The EMPIAR 10643 dataset corresponds to a cryo-ET acquisition of the HIV-1 GagdeltaMASP1T8I assemblies. It was acquired with an angular range from −60° to 60°, and with an angular increment of 3°. The EMPIAR 10453 dataset is a cryo-ET acquisition of SARS-COV-2. It was acquired in the same conditions as the previous dataset. The EMPIAR 11462 dataset is a cryo-ET acquisition of an ER-mitochondria encounter structure in cryo-FIB milled yeast cells. It was acquired with an angular range from −56° to 56° at 1° increment. These three datasets have been acquired several times: 5 times for the first dataset, 266 for the second, and 51 for the third. Nevertheless, in our reconstruction only one series will be used at a time. In the following, we will reconstruct one series from EMPIAR 10453 dataset, two separate series from EMPIAR 10643: the Series 40 and 51 (we consider as different datasets), and one from EMPIAR 11462.

### 3.2 Data preprocessing

The three used datasets have embedded fiducial markers to perform the alignment. Before applying the different reconstruction algorithms, we preprocess the projections as follows: first, we completed an alignment of the projections using IMOD software (Mastronarde and Held 2017). Then, we padded the missing area due to the alignment, using a gray value equal to the average over the projections. For the reconstructions, we applied masks to disable the missing area and the fiducial markers. The input projections have 4K (4096×4096) or even 8K (8192×8192) resolutions. For computational convenience, we downsampled the projections by a factor of five for EMPIAR 10453 and EMPIAR 10643, and eight for EMPIAR 11462 to get a resolution close to 1024×1024. We also cropped them to have a better focus on the virus copies while keeping the same size for all datasets.

### 3.3 Evaluation of the denoising effectiveness

**Figure 5.**
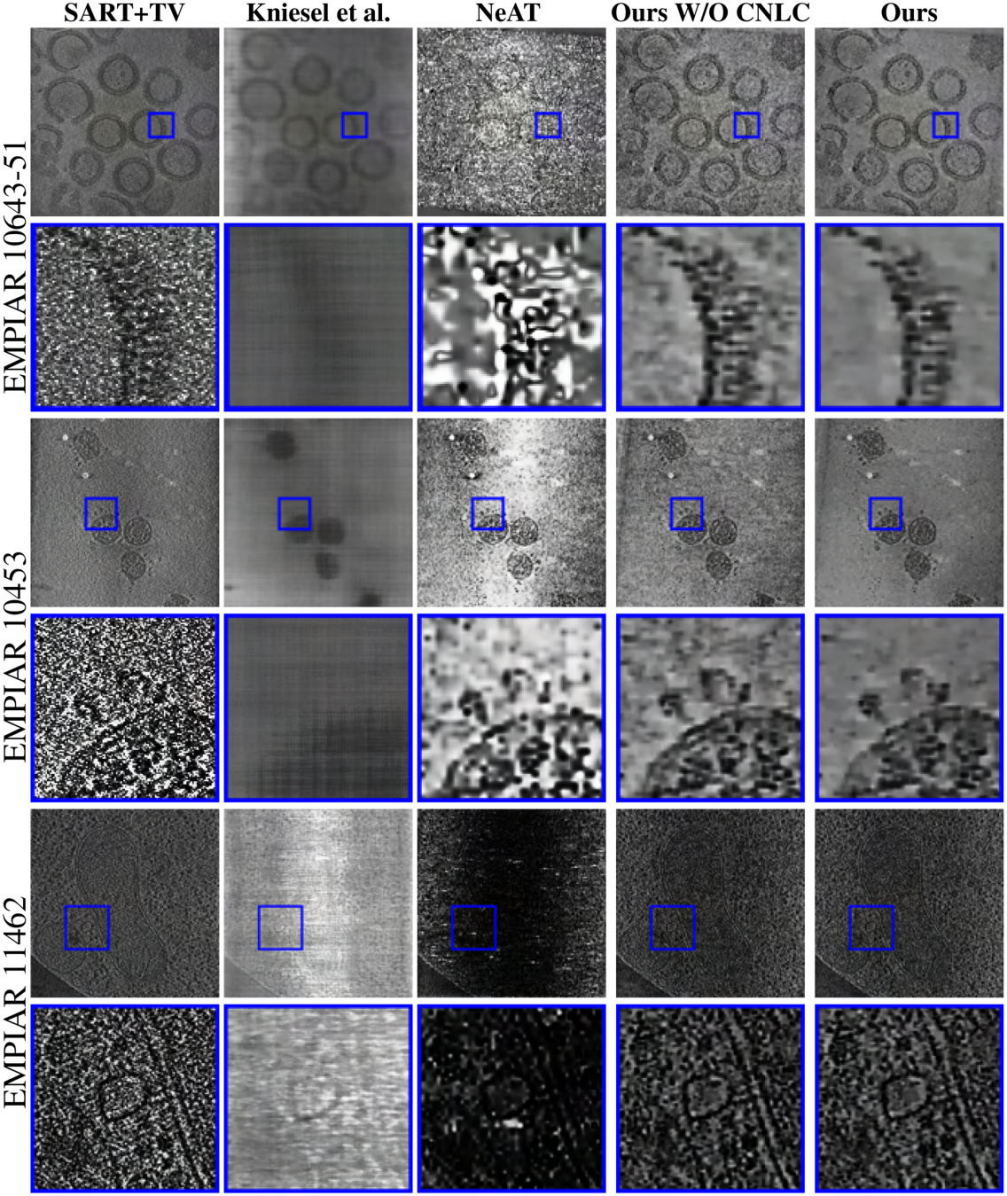
Reconstruction results using different methods of two datasets, with a zoom on detailed features of the viruses or cells: SARS-COV-2 (EMPIAR 10643-51), HIV-1 (EMPIAR 10643), and ER-mitochondria encounter structure in cryo-FIB milled yeast cells (EMPIAR 11462).

In Fig. 5, we show a comparison of the reconstructed datasets, using the different reconstruction methods previously introduced. Globally, all methods except the **Kniesel *et al.* method** yield a similar contrast on the reconstructed volume. This could be explained by the difference between the datasets on which this approach was learned and the real data used in this comparison. In the following, we will focus our comparison on the effectiveness of the approaches in denoising the data, and their ability to save the interesting features in the reconstruction.

From Fig. 5, we can see that our approach is the most effective in suppressing noise in uniform regions. Even the use of the TV constraint **SART+TV** and **NeAT** reconstructions still contain noise in the uniform regions. If the weight of this constraint is increased to improve the denoising power of **SART+TV** and **NeAT**, the main features in the reconstructions will be lost. The denoising effectiveness of our approach in comparison to **NeAT** can be explained not only by the use of the CNLC but also by our octree-update strategy. Indeed, **NeAT** updates the octree based on the reprojection error. However, in the cryo-ET datasets, the noise is very high. It is then hard to separate the noise from the reprojection error. Moreover, we used a multi-scale strategy to update the octree structure in our approach (see Section 2.6.1).

Furthermore, we report in Table 1 three statistical metrics: the Contrast-to-Noise Ratio (CNR), the Equivalent Number of Look (ENL), and the signal-to-noise ratio (SNR), which are commonly used to evaluate the denoising quality (Li *et al.* 2017, Bepler *et al.* 2020). These three metrics are defined as follows:


(11) $$\text{CNR}=\frac{1}{N_{pr}}\sum \frac{\mu_{f}-\mu_{u}}{\sqrt{0.5(\sigma_{f}^{2}+\sigma_{u}^{2})}},$$

where $N_{pr}$ is the number of paired regions selected to compute the CNR. $\mu_{f}$ and $\sigma_{f}^{2}$ refer to the mean and variance in the selected regions containing features. $\mu_{u}$ and $\sigma_{u}^{2}$ are the mean and variance in the selected uniform regions. We illustrate these selected regions in the Supplementary Material.


(12) $$\text{ENL}=\frac{1}{N_{r}}\sum \frac{\mu_{r}^{2}}{\sigma_{r}^{2}},$$

where $N_{r}$ is the number of regions selected to compute the ENL. $\mu_{r}$ and $\sigma_{r}^{2}$ refer to the mean and variance in the selected homogeneous regions.

**Table 1.** Evaluation of the contrast enhancement (CNR), the smoothing (ENL), and the denoising effect (SNR) obtained using the different methods (best results highlighted in bold).

Metric	Dataset	SART+TV	NeAT	Ours ($L_{\mathrm{data}}$)	Ours ($L_{\mathrm{data}}$, $L_{tv}$)	Ours W/O CNLC	Ours
CNR ↑	EMPIAR 10643-51	0.141	0.283	0.273	0.375	0.377	0.541
	EMPIAR 10453	0.049	0.444	0.207	0.431	0.431	0.715
ENL↑	EMPIAR 10643-51	22.311	15.822	28.981	64.440	65.841	282.164
	EMPIAR 10453	6.708	47.706	32.373	88.132	89.545	496.075
SNR ↑	EMPIAR 10643-51	−10.748	−10.956	−5.015	−1.288	−1.116	3.095
	EMPIAR 10453	−25.190	−2.988	−9.975	−8.541	−8.231	1.527

Following the method proposed in Bepler *et al.* (2020), we manually label signal and background regions on different slices of the reconstructed tomogram, and then compute the mean SNR (in dB) using this formula:


(13) $$\text{SNR}=\frac{10}{N_{pr}}\sum {\;\text{log}\;}_{10}\left({\left(\mu_{f}-\mu_{u}\right)}^{2}\right)-{\;\text{log}\;}_{10}(\sigma_{u}^{2}).$$

Notations are the same as for the CNR metric.

The CNR evaluates how the denoiser strategy increases the contrast between the ROI and the uniform background, while ENL measures the smoothness in the homogeneous areas. The SNR metric measures the signal noise ratio (denoising effect) in the reconstruction. In Table 1, we did not include the **Kniesel *et al.*** approach because it is not well adapted in the reconstruction of the used real datasets, as mentioned before. The results of the table show higher CNR, ENL, and SNR values with our approach, which confirms the qualitative observations. Our approach increases the contrast between the regions with features and the background, and yields smoother uniform regions. From this table, one can notice the huge contribution of the CNLC in improving the denoising effect of our approach. This table also shows that the **SART-TV** result is the noisiest.

### 3.4 Detailed feature analysis

Figure 5 shows that **SART+TV** is good at preserving the detailed features. However, when we zoom in on those features, they appear flooded in the background noise. It is then hard to use this approach to analyze the structure of the features. The **NeAT** reconstruction technique preserves relatively well the structure of the viruses in the reconstruction of EMPIAR 10453, even if there is a non-uniform contrast level in the reconstruction. However, in the EMPIAR 10643 dataset, this approach’s result is highly impacted by the noise. The features are hardly distinguishable from the noisy background. Finally, this figure illustrates a better performance using our reconstruction in preserving the detailed features in both datasets. Without the use of the non-local constraint, the result contains the main features of the datasets, but also some residual noise. When using the non-local prior, this residual noise is reduced. As a result, the structure of the viruses is easily distinguishable from the uniform background.

**Figure 6.**
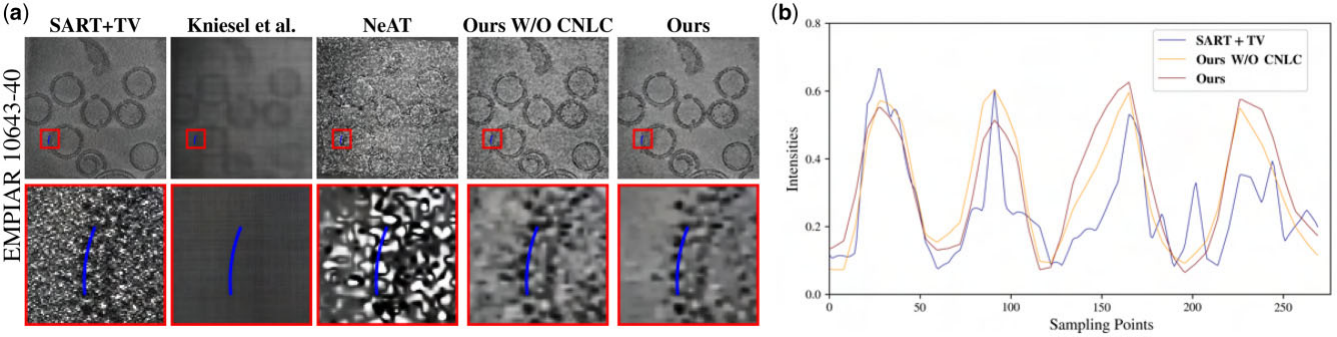
Reconstruction results and profile analysis of the EMPIAR 10643-40 dataset. (a) Reconstruction results of EMPIAR 10643-40 using different methods, with a zoom on the spikes of the SARS-COV-2. (b) Intensity profile along the curve in blue (zoomed region) for different reconstruction of EMPIAR 10643-40 dataset.

To numerically evaluate the performance of each approach in recovering detailed features, we analyze the profile of the reconstructions along a line around the viruses from the EMPIAR 10643-40 dataset, as shown in Fig. 6a. This line was selected and sampled manually, in order to highlight the performance of each method in preserving the periodic structure of the spikes of the virus. Figure 6a shows that the **Kniesel *et al.*** and **NeAT** do not allow the separation between the spikes and the background, for this dataset. Therefore, we focus profile comparison on the remaining approaches. The selected line intersects with five spikes having low intensities. It also intersects with background intervals between successive spikes. The expected profile should look like a smooth periodic function, alternating between peaks (background) and valleys (spikes).

Figure 6b represents the profiles for the three methods compared: **SART+TV**, **Ours W/O CNLC**, and **Ours**. The profile of the **SART+TV** approach is less regular than the two others. We can notice some intermediate peaks and valleys caused by the residual noise in the reconstruction. It is also hard to use this profile to localize exactly the position of the spikes. On the other hand, using our approach with or without the non-local constraint yields a more regular profile, where the spikes and the background can be separated with a simple threshold filtering. From this profile analysis, the impact of the non-local constraint seems to be minor. Indeed, this prior is more adapted to reduce the noise in the uniform regions, where several areas through the volume have the same statistics. For the virus areas, only a few regions have similar statistics. Our implementation of this prior reduces its impact on such regions. Since we select a random pair of density grids each time, we will likely get uniform density grids or dissimilar parts of the virus.

### 3.5 Resolution evaluation

To evaluate the precision of a reconstruction technique, the FSC is widely used in Single Particle Analysis. It is computed from two different reconstructions of the same structure as follows:


(14) $$\text{FSC}(r_{i})=\frac{\sum_{r_{i}}F_{1}(r_{i})F_{2}{\left(r_{i}\right)}^{*}}{\sqrt{\sum_{r_{i}}|F_{1}(r_{i}){|}^{2}\sum_{r_{i}}|F_{2}(r_{i}){|}^{2}}},$$

where $r_{i}$ is the voxel element in Fourier space at radius *r*, $F_{1}(r_{i})$ and $F_{2}(r_{i})$ are the complex structure factors of the first and second volume, respectively. In tilt-series cryo-ET, we have only one copy of the reconstructed volume. Therefore, to compute the FSC in this case, Diebolder *et al.* (2015) suggested to split the even and odd projections to reconstruct two different volumes of the same sample. To evaluate the reconstruction resolution, we followed this approach and evaluated the FSC on the EMPIAR 10643-40, and EMPIAR 11462 datasets.

The reconstruction resolution is defined as the highest frequency where the FSC remains above a given threshold (0.5 and 0.143 are commonly selected). In our case, we used FSC=0.5, because with FSC=0.143, we cannot distinguish the resolution for some reconstruction methods. The curves of the computed FSC are illustrated in the Supplementary Material for both datasets. They show that the amplitudes of high spatial frequencies are more “consistent” across the two reconstructions obtained from the odd and even projections when our method is used for reconstruction versus when the comparison methods.

In Table 2, we report the obtained quantitative resolution estimates in Å obtained for the different compared approaches. Although this analysis produces the best resolution estimates for our method, we caution against over-interpreting the numerical FSC values due to the high residual noise levels in the reconstructions based on the further reduced number of projections used to calculate the metric. Note that the FSC would rank methods highly that produce consistent but wrong structures across the two reconstructions. Therefore, unlike single particle cryo-EM, we believe that for tilt-series cryo-ET the numerical value of the FSC metric does not give a reliable estimate of the absolute resolution. With this in mind, the results do seem to corroborate the qualitative analysis that we can draw from the results shown in Fig. 5.

**Table 2.** Evaluation of the reconstruction resolution (in Å) from FSC=0.5 for different reconstruction methods (best results highlighted in bold).

Dataset	SART+TV	Kniesel *et al.*	NeAT	Ours W/O CNLC	Ours
EMPIAR 10643-40	634.8	2948.9	755.7	663.0	**600.3**
EMPIAR 11462	239.9	578.8	896.3	221.6	**203.7**

## 4 Conclusion

In this article, we presented a new adaptive tomography reconstruction method for tilt-series cryo-ET data. This approach uses an octree structure, where each octant stores a 3D density grid. During the optimization step, we first update the structure and the 3D density grids using a down-sampled version of the projections. Then, we freeze the octree and fine-tune the density grids using the original projections. Our loss function includes the image formation model, a TV prior, a cross-octants non-local constraint, and an octant-boundary consistency prior.

The qualitative and quantitative evaluations of our approach on real dataset show a better reconstruction quality of our approach in comparison to the state-of-the-art methods. By using the differentiable grid, we can overcome the artifacts caused by the missing-wedges acquisition. On the other hand, the octree structure allows a better handling of the large amount of data in a reasonable time. Meanwhile, the three constraint terms of the loss considerably reduce the noise level. However, a parameter tuning is essential to avoid signal loss due to over-denoising.

Our current framework is now limited to 1K resolution of projections. Further computation reduction, both in memory and time, will be a potential trend that we will investigate in the future. We think that through the reduction, we could reconstruct higher resolution volumes. In addition, taking the alignment into account, we will also consider a joint alignment and reconstruction approach.

## Supplementary Material

vbad131_Supplementary_Data

## Data Availability

All experimental data in this article are available at EMPIAR website.
