# Supplementary material for: Adaptive differentiable grids for cryo-electron tomography reconstruction and denoising
Source: Bioinform Adv. 2023 Sep 22;3(1):vbad131. doi: 10.1093/bioadv/vbad131 (PMC10560095; doi:10.1093/bioadv/vbad131)
Supplement: vbad131_Supplementary_Data [file vbad131_supplementary_data.pdf]

Adaptive Differentiable Grids for Cryo-Electron Tomography Reconstruction and Denoising
— Supplementary —

Yuanhao Wang[1], Ramzi Idoughi[1], Darius Rückert[2], Rui Li[1], and Wolfgang Heidrich[1,*]

[1]Visual Computing Center (VCC), King Abdullah University of Science and Technology (KAUST), Thuwal, 23955-6900, Saudi Arabia
[2]Department of Computer Science, Friedrich-Alexander-Universität Erlangen-Nürnberg (FAU), Schlossplatz 9, 91054, Erlangen, Germany

1 Implementation Details

We fully implemented our framework in C++. During our validation experiments, we ran our framework on two different workstations: both are equipped with the processor Intel(R) Xeon(R) Gold 6242 CPU, and have 512 GB memory. The first workstation is equipped with Nvidia RTX 8000s, while the other has an Nvidia A6000s GPU. Both of these workstations were running on Ubuntu 18.04 LTS.

During the optimization of the density grids, we took the root mean squared propagation optimizer (RMSProp), and defined the learning rate as 0.01.
The convergence is obtained after 25 epochs, where an epoch is here defined as one pass through all the available projection data. During the first 15 epochs, the octree is updated using the downsampled projections described in the main paper. Then, during the remaining epochs, the octree structure is fixed, and only the differentiable density grids are fine-tuned using the original projections, which improves the reconstruction. We set the maximum number of octree-nodes (that stores the density grids) to be equal to 320. The grid size depends on the final volume size to be reconstructed. Here, we took the grid size to be equal to $42 \times 42 \times 42$ in real scenes and $24 \times 24 \times 24$ for synthetic data.

After a parameter tuning search we found that setting $\lambda_{bc} \in \left[10^{-4}, 4 \cdot 10^{-4}\right]$ will give better boundary consistency in all cases, and λ_{tv} is also a parameter that could be selected in a range of $\left[10^{-4}, 6 \cdot 10^{-4}\right]$. While only adding a small weight for the CNLC (for example, $\lambda_{cnlc} = 10^{-6}$) will produce good denoising.

1.1 Mask generation

For each projection we design a mask to exclude the fiducial markers used for the alignment. In our approach the alignment is a preprocessing step, that we perform

using IMOD. Thus, the positions of the fiducial beads is contained in the "*.fid" files, output by IMOD. We run the *model2point* command of IMOD to convert it into a ".txt" file, containing the markers' positions. At this stage, we double-check that all markers, inside the region of interest, are detected. For some datasets, the input projections are already aligned. In this case, we use the *imodfindbeads* command in IMOD to detect the beads, and retrieve their positions as above.
We also exclude from our mask the padding regions, to keep only the region of interest. This step could be undertaken automatically if we know the alignment matrix and the original projection by coding. Then our final masks are obtained by merging the masks on fiducial markers and those on padding regions, as shown in Figure 1.

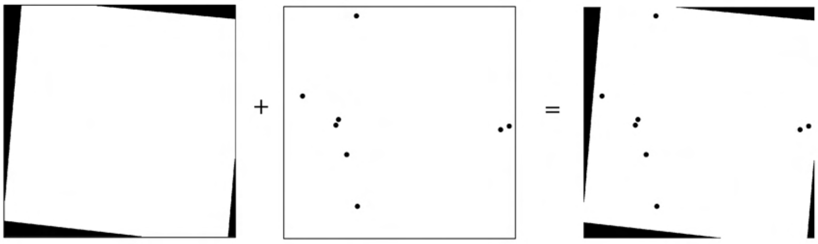

Figure 1: Mask generation

2 Experiments on synthetic dataset

The synthetic dataset used in our experiments is similar to the one proposed in Kniesel et al. [2022]. It corresponds to randomly distributed ellipsoidal shells with random densities, that reproduce the density model of the ZIKV (i.e., Zika) virion at 15 Å Long et al. [2019]. The 3D volume is then projected in the angular range of $[-70°, 69.5°]$, with an angular step of $1.5°$. Gaussian noise is finally added to the obtained projections. Several noise levels have been tested. When the STD of the noise is higher than 0.05 (see Section 2.2), the obtained projections look visually close to the real captured cryo datasets, in terms of the noise. In the following, we present the results of our studies using the synthetic data, where we compare the performances of our method with different grid sizes and in the presence of different noise levels.

2.1 Impact of the grid size

As described in the density grids optimization, the grid size $N_x \times N_y \times N_z$ with $N_x = N_y = N_z$ defines the number of blocks stored inside each octree node (i.e., density grid). For smaller grid sizes, the training is faster, and the reconstructed volume is smoother and cleaner. However, the grid is not efficient enough to learn detailed features. On the other hand, with large grid sizes, more details are recovered, but the reconstruction is prone to overfitting and reproducing noise. This is well illustrated in the Figure 2. In this figure, the zoom of the blue frame shows a detailed feature, while the zoom of the red frame shows uniform regions of the sample. One can notice that with the smallest grid size (12), the uniform regions are well retrieved, but the star in the blue frame is blurred. With the largest grid size (34), the star is relatively well retrieved with low blur effect. However, the uniform regions contain more noise than the same regions for

the other grids. A compromise should be found for a better recovering of the detailed features, while removing as much noise as possible.

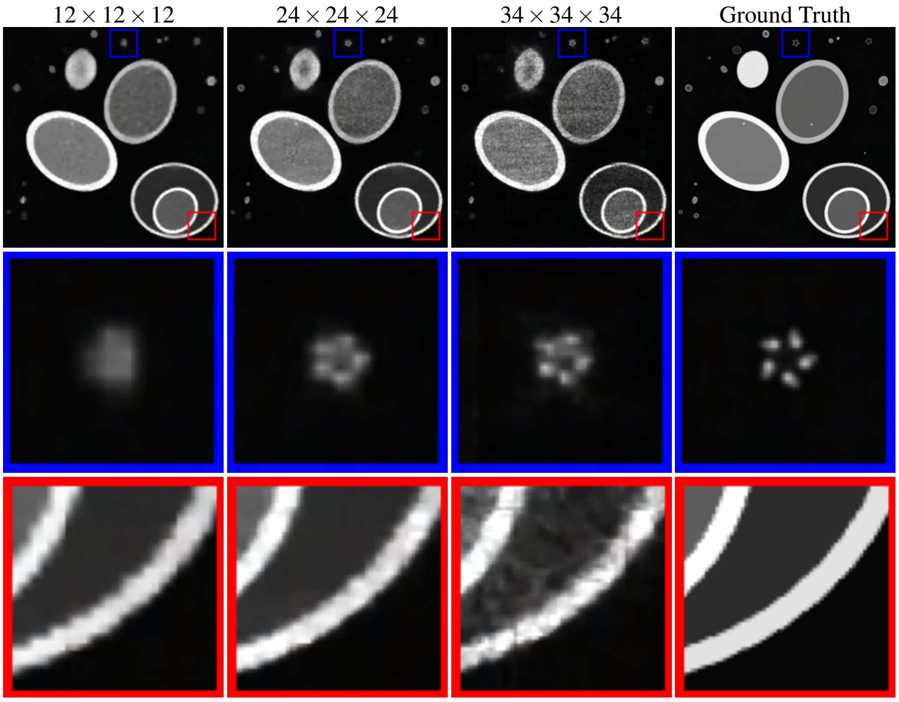

Figure 2: Comparison of the impact of different grid sizes on the reconstruction of the synthetic dataset.

We also conducted a quantitative evaluation of the Peak Signal-to-Noise Ratio (PSNR) and Structural Similarity Index Measure (SSIM) of the reconstructed volume for different grid sizes. The PSNR is given as follows:

$$\mathrm{PSNR} = 20\log_{10}\left(\frac{\max(g)}{\sqrt{\mathrm{mean}\|r-g\|_2^2}}\right) \tag{1}$$

The SSIM is formulated as:

$$\mathrm{SSIM}(r,g) = \frac{(2\mu_r\mu_g + c_1)(2\sigma_{rg} + c_2)}{(\mu_r^2 + \mu_g^2 + c_1)(\sigma_r^2 + \sigma_g^2 + c_2)} \tag{2}$$

where g represents the ground truth of the volume, and $\max(g)$ is the maximum value in the volume, r is the reconstructed volume. μ_r and σ_r^2 refer to the mean and variance in the reconstructed volume. μ_g and σ_g^2 refer to the mean and variance in the ground truth volume. σ_{rg} is the covariance of the reconstructed volume and the ground truth volume. c_1 and c_2 are two small variables to stabilize the division.
Note that the SSIM is evaluated only on a region containing detailed features, as shown in the second line of the Figure 2.

In this experiment, we set the standard deviation of the noise to 0.5. We represent, in Table 1, the PSNR and SSIM for different grid sizes. We compared our approach

with and without using the non-local constraint. Globally, we notice good PSNRs (higher than $39.5dB$) for all the grid sizes. The best PSNRs are obtained for grid sizes (N_x, N_y, N_z) between $[16, 22]$. However, the SSIM is the maximum for a grid size equal to 24, which seems to be the best compromise in terms of recovering details and removing noise. In the following, we will use this grid size for evaluations on the synthetic dataset.

Table 1: Quantitative evaluation of the impact of the grid size(larger is better)

Grid Size \ Method	**Ours W/O CNLC**		**Ours**	
	PSNR (dB) ↑	SSIM ↑	PSNR (dB) ↑	SSIM ↑
$12 \times 12 \times 12$	42.0416	0.8658	41.7882	0.8590
$14 \times 14 \times 14$	42.2870	0.8938	42.1812	0.8819
$16 \times 16 \times 16$	**42.3510**	0.9027	42.3725	0.8991
$18 \times 18 \times 18$	42.2464	**0.9182**	**42.4315**	0.9160
$20 \times 20 \times 20$	42.0243	0.9121	42.3148	0.9182
$22 \times 22 \times 22$	41.6389	0.9066	42.1616	0.9150
$24 \times 24 \times 24$	41.1991	0.9027	41.7771	**0.9228**
$26 \times 26 \times 26$	40.7861	0.8927	41.4353	0.9102
$28 \times 28 \times 28$	40.3329	0.8836	41.0690	0.9034
$30 \times 30 \times 30$	39.8482	0.8807	40.5566	0.9021
$32 \times 32 \times 32$	39.3598	0.8751	40.0843	0.8921
$34 \times 34 \times 34$	38.8721	0.8610	39.5352	0.8830

2.2 Impact of the noise level

In this experiment, we compare the performance of our approach to different baseline methods, for different noise levels. The first baseline is (**SART + TV**), a well-established iterative reconstruction technique (SART) combined with a total variation prior. We also used two recent neural based approaches: **Kniesel et al.** Kniesel et al. [2022], and **NeAT** Rückert et al. [2022]. We also report the results of our reconstruction without using the non-local constraint. For a fair comparison, all the output densities have been normalized into the same range $[0, 1]$.

In the Figure 3, we show the results obtained with the compared methods for two different noise levels: 0.02 and 0.05. Similarly to the previous experiment, we provide a zoom on two regions in the dataset: the blue frame for the detailed feature, and the red frame for the uniform region. As we can see in this figure, **SART+TV** can recover the details poorly, but it maintains a lot of noise even for low noise levels. **Kniesel et al.** approach is good in denoising the reconstruction when the noise level is low, but it is not robust to high noise levels. Moreover, the details are poorly retrieved even for a low noise level. **NeAT** is relatively good at preserving the details, even for the higher noise level. However, it is not robust for denoising high levels of noise. On the other hand, our approach (**Ours**) has better robustness in denoising uniform regions, while it has a good recovering of features. The impact of the non-local constraint can be clearly seen in denoising uniform regions in the case of high noise levels.

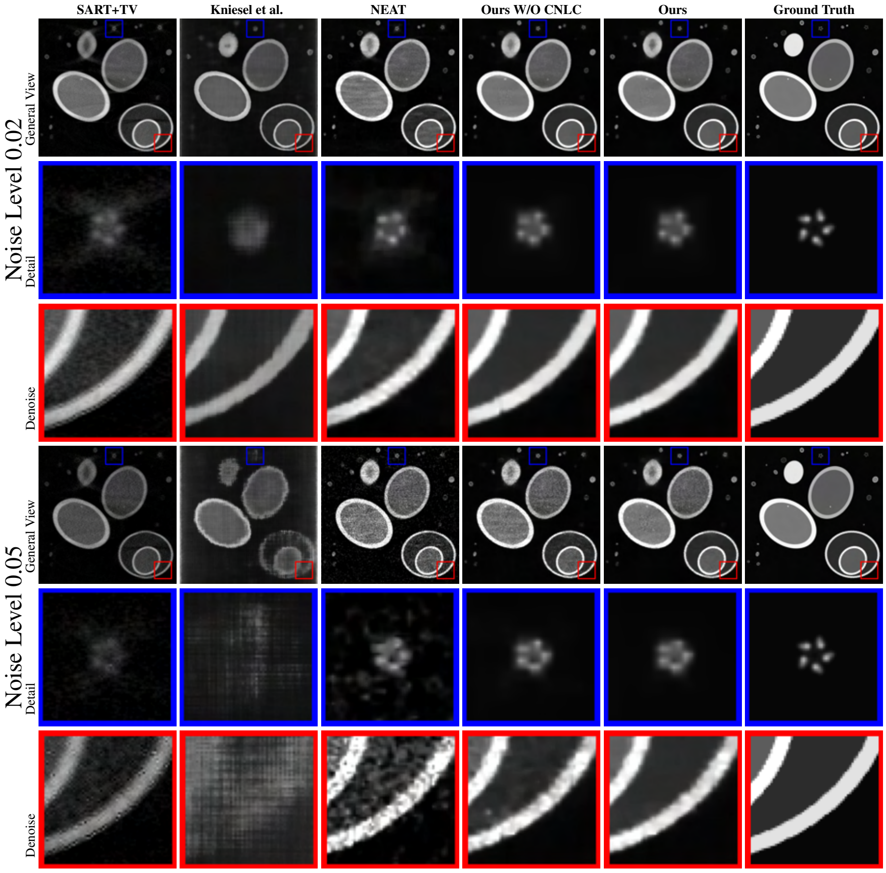

Figure 3: Comparison of reconstruction results using different methods. From left to right: **SART+TV Kniesel et al.**, **NeAT**, **Ours W/O CNLC** and **Ours**. We depicted two different noise levels and zoomed in to view the details and denoise effect visually.

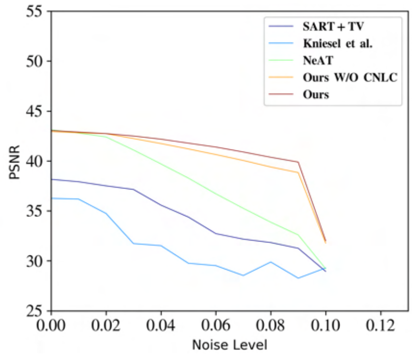

(a) PSNR comparison for different noise levels

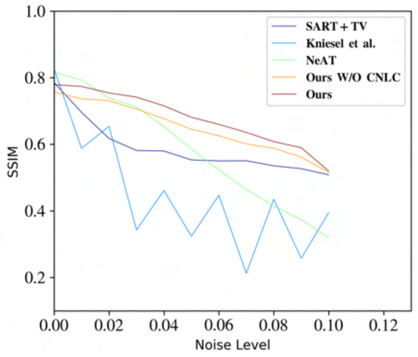

(b) SSIM comparison for different noise levels

Figure 4: Comparison of the PSNR/SSIM obtained with different reconstruction methods for different noise levels.

3 Additional experiments using real datasets

3.1 Validation of the TV prior

Figure 5 illustrates the impact of TV prior on the quality of the tomogram reconstruction. We ran our validation on EMPIAR 10643-40, and we can see apparent quality improvement with **TV** prior as a base prior.

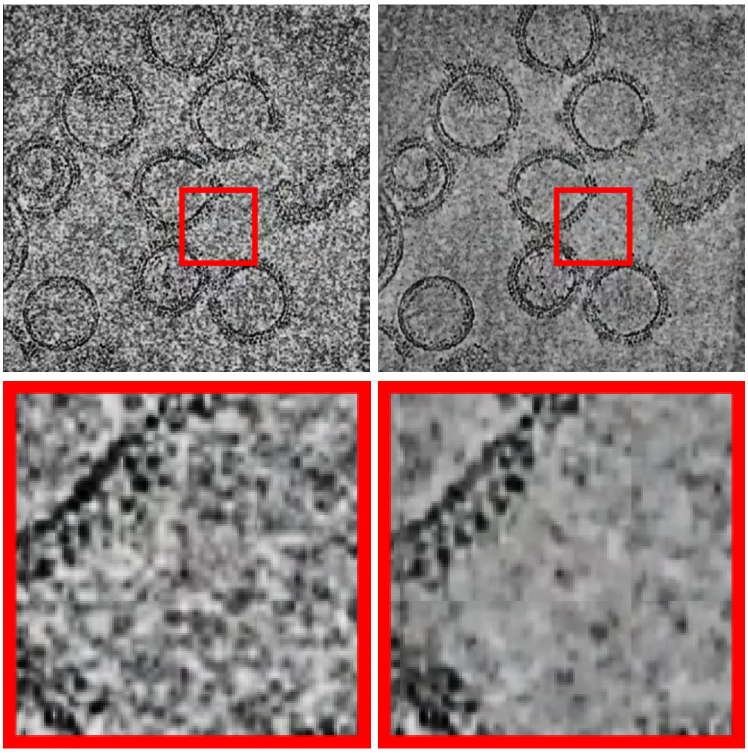

Figure 5: Comparison of a reconstruction using only $\mathscr{L}_{data}$ in the loss function (left side), with another that uses both $\mathscr{L}_{data}$ and $\mathscr{L}_{tv}$ (right side)

3.2 Validation of the BC prior

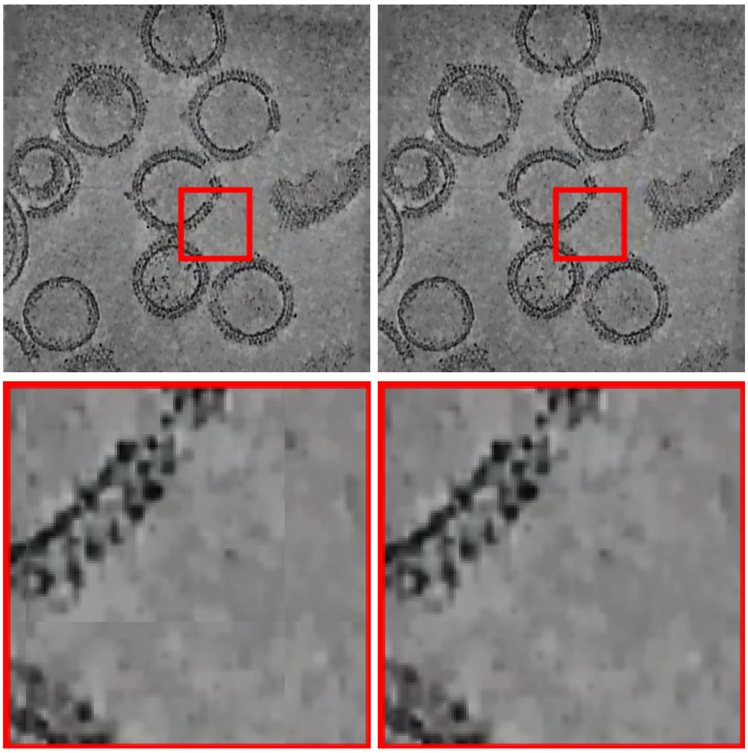

Figure 6: Comparison of a reconstruction that does not involve the edge preservation constraint (left side), with another that uses the **BC** prior (right side). The edge preservation constraint removes the block-like artifacts and improves the reconstruction quality.

Figure 6 illustrates the impact of BC prior on the quality of the tomogram reconstruction. We ran our validation on EMPIAR 10643-40, and we can see apparent boundary discontinuity without **BC** prior. When we use **BC** prior, the artifacts are completely removed.

3.3 Validation of the CNLC prior

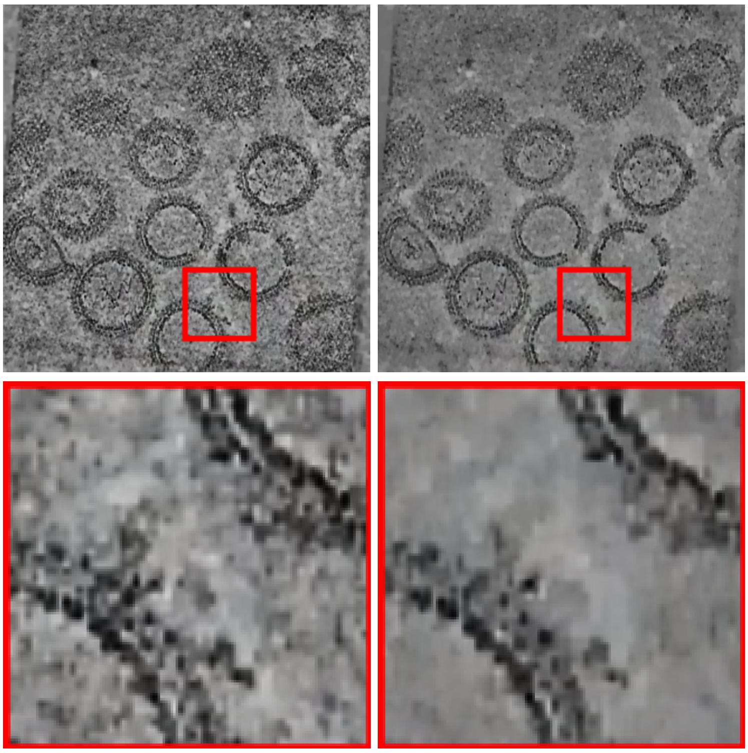

Figure 7: Comparison of a reconstruction that does not involve the non-local constraint (left side), with another that uses the non-local constraint (right side). The non-local loss helps denoise the reconstructed tomogram, while saving most of the important features.

To validate the effectiveness of the CNLC regulariser on the final result, we illustrate in the Figure 7 a slice visualization of the reconstructed tomogram using the CNLC (right side), and the same sample reconstructed without using this prior (left-side). One can see clearly the denoising effect of this non-local prior.

3.4 Selection of CNR and ENL regions

In the main paper we introduce the CNR and ENL metrics to evaluate the effectiveness of our method in denoising the reconstructed tomogram. We illustrate in this paragraph how to select the regions to compute these two metrics. Indeed, we manually select CNR region pairs and ENL regions, and apply the selection to all the comparing methods. Here to compare the volume, we use 3D metrics by adding a depth of 10 to the selected rectangle planes as depicted in Figure 8.

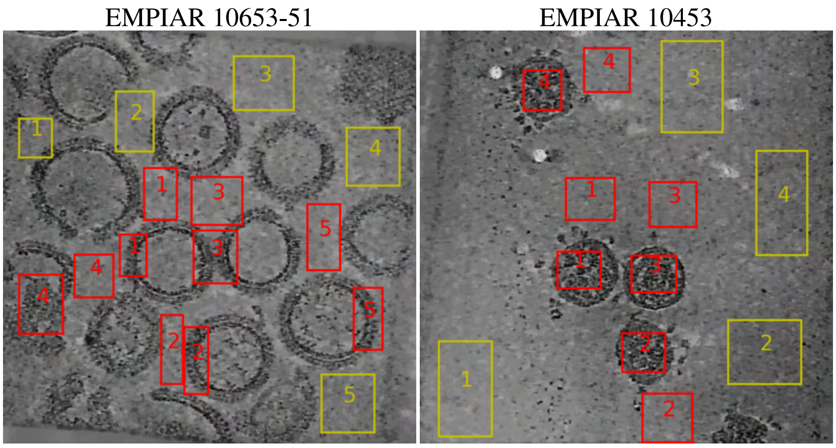

Figure 8: CNR region pairs and ENL region selected for EMPIAR 10643-51 and EMPIAR 10453. The CNR region pairs, one with features and the other with background noise, are represented by red rectangles. The chosen homogeneous areas used for computing the ENL are represented by yellow rectangles.

3.5 Evaluation of the reconstruction resolution

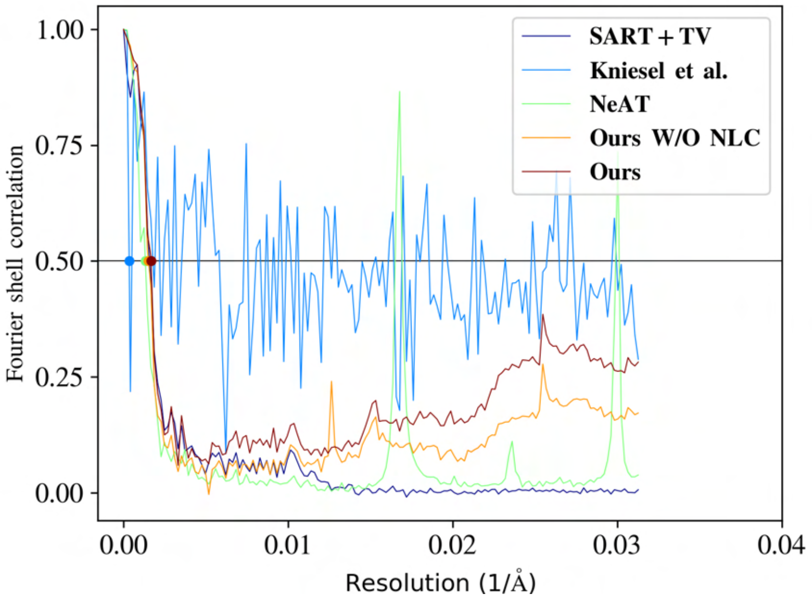

Figure 9: FSC computed for the reconstruction of EMPIAR 10643-40 dataset using different methods

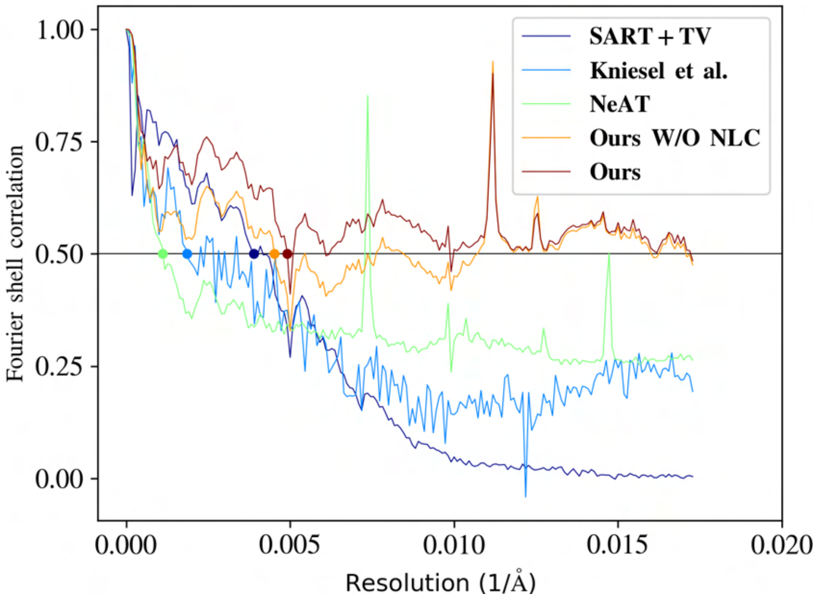

Figure 10: FSC computed for the reconstruction of EMPIAR 11462 dataset using different methods

In Figure 9 and Figure 10 we illustrate the result obtained when computing the FSC

for the different compared method on the EMPIAR 10643-40 and EMPIAR 11462 datasets respectively.

3.6 Slice View of EMPIAR 10643-40

We visualize the reconstruction slices from EMPIAR 10643-40 using our method. Our method can denoise the whole volume, as seen from these slices.

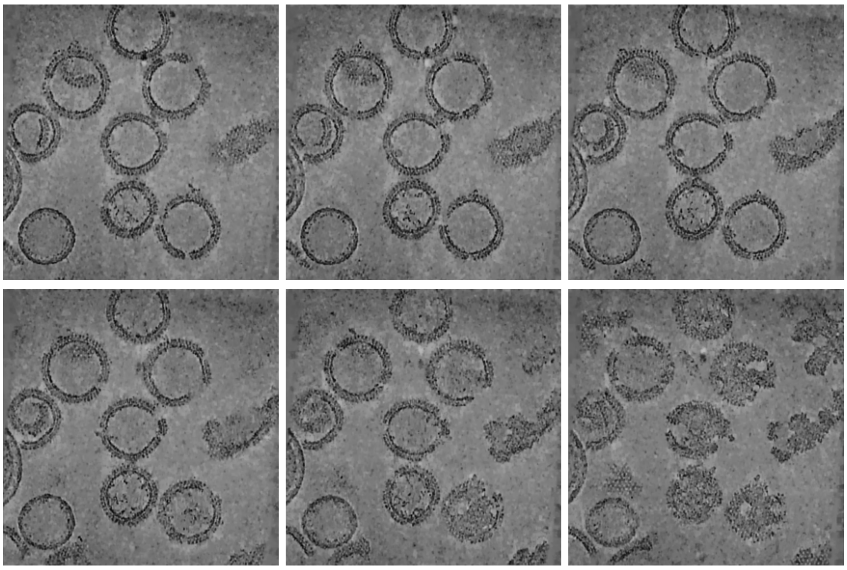

Figure 11: Reconstruction slices from EMPIAR 10643-40 with our methods.

4 Number of parameters and Running time

The MLP-based representation used in **Kniesel et al.** approach requires around 2.7 M parameters, **NeAT** 168 M parameters, while **Ours** uses 28 M parameters. Even if our approach uses 10 times more parameters than MLP-based approaches, it has a faster convergence and remains manageable for the GPUs.

We compare in the Table 2 the total execution time needed to perform the training /optimizing and reconstruction using each approach. In this table, we did not report the running time of the **SART+TV**, because this approach is implemented and accelerated using OpenMP, and runs on CPU. It lasts around one hour to converge, when using our workstations Intel(R) Xeon(R) Gold 6242 CPUs, with 64 threads. From this table, we can notice that the non-local constraint represents around 66% of the execution time in our approach. Nevertheless, even when using this constraint, our approach remains slightly faster than **NeAT**. **Kniesel et al.** is the slowest approach.

Table 2: Comparisons of running times (hours) used for the different reconstruction methods.

Method	GPU	**Kniesel et al.**	**NeAT**	**Ours W/O CNLC**	**Ours**
Synthetic	RTX A6000	100.65	0.62	0.2	0.6
EMPIAR 10643 (40 and 51)	RTX A6000	33.45	0.78	0.19	0.56
EMPIAR 10453	RTX 8000	33.03	0.65	0.24	0.63

References

H. Kniesel, T. Ropinski, T. Bergner, K. S. Devan, C. Read, P. Walther, T. Ritschel, and P. Hermosilla. Clean implicit 3D structure from noisy 2D STEM images. In *Proc. CVPR*, pages 20762–20772, 2022.

F. Long, M. Doyle, E. Fernandez, A. S. Miller, T. Klose, M. Sevvana, A. Bryan, E. Davidson, B. J. Doranz, R. J. Kuhn, et al. Structural basis of a potent human monoclonal antibody against zika virus targeting a quaternary epitope. *Proceedings of the National Academy of Sciences*, 116(5):1591–1596, 2019.

D. Rückert, Y. Wang, R. Li, R. Idoughi, and W. Heidrich. NeAT: Neural Adaptive Tomography. *ACM Trans. Graph.*, 41(4), July 2022.
